# Discovery of a New Starship Transposon Driving the Horizontal Transfer of the *ToxA* Virulence Gene in *Alternaria ventricosa*

**DOI:** 10.3390/microorganisms13020376

**Published:** 2025-02-09

**Authors:** Fei Liu, Ratchadawan Cheewangkoon, Rui-Lin Zhao

**Affiliations:** 1 State Key Laboratory of Mycology, Institute of Microbiology, Chinese Academy of Sciences, Beijing 100101, China; liuf@im.ac.cn; 2 Department of Entomology and Plant Pathology, Faculty of Agriculture, Chiang Mai University, Chiang Mai 50200, Thailand; 3 College of Life Science, University of Chinese Academy of Sciences, Beijing 100049, China

**Keywords:** *Alternaria ventricosa*, horizontal gene transfer, *ToxA*, transposon

## Abstract

The virulence gene *ToxA* has been proposed to be horizontally transferred between three fungal wheat pathogens (*Parastagonospora nodorum*, *Pyrenophora tritici-repentis*, and *Bipolaris sorokiniana*) as part of a conserved ~14 kb *ToxhAT* transposon. Here, our analysis of 2137 fungal species-representative assemblies revealed that the *ToxA* gene is an isolate of *Alternaria ventricosa* and shows a remarkable 99.5% similarity to those found in *B. sorokiniana* and *P. tritici-repentis.* Analysis of the regions flanking *ToxA* within *A. ventricosa* revealed that it was embedded within a 14 kb genomic element nearly identical to the corresponding *ToxhAT* regions in *B. sorokiniana*, *P. nodorum*, and *P. tritici-repentis*. Comparative analysis further showed that *ToxhAT* in *A. ventricosa* resides within a larger mobile genetic element, which we identified as a member of the Starship transposon superfamily, named *Frontier*. Our analysis demonstrated that *ToxhAT* has been independently captured by three distinct Starships—*Frontier*, *Sanctuary*, and *Horizon*—which, despite having minimal sequence similarity outside of *ToxhAT*, facilitate its mobilization. These findings place *Frontier*, *Sanctuary*, and *Horizon* within a growing class of Starships implicated in the horizontal transfer of adaptive genes among fungal species. Moreover, we identified three distinct HGT events involving *ToxA* across these four fungal species, reinforcing the hypothesis of a single evolutionary origin for the *ToxhAT* transposon. These findings underscore the pivotal role of transposon-mediated HGT in the adaptive evolution of eukaryotic pathogens, offering new insights into how transposons facilitate genetic exchange and shape host–pathogen interactions in fungi.

## 1. Introduction

Horizontal gene transfer (HGT), the non-Mendelian exchange of genetic material between organisms, is a powerful force driving rapid adaptation, especially in microbial pathogens [1,2]. By enabling the acquisition of new traits, HGT can significantly enhance virulence and antibiotic resistance, expand host ranges, and improve competitive abilities in various environments [3,4,5]. Although HGT was once considered rare in eukaryotes, its impact on adaptation, particularly among microbes sharing hosts, is now increasingly recognized [1]. Fungi, with their relatively small genomes, critical roles in human and plant diseases, and extensive use in food and biotechnology, have emerged as ideal models for studying eukaryotic adaptation through HGT [6,7]. Recent studies have highlighted the widespread occurrence of HGT in fungi [8,9], even in complex multicellular forms like mushrooms [10]. Notably, the discovery of large transposable elements in fungal genomes, many of which harbor genes involved in HGT, underscores the crucial role these elements play in fungal evolution [11,12].

A prime example of HGT in fungi is the necrotrophic effector *ToxA*, initially identified in the wheat pathogen *Pyrenophora tritici-repentis*, where it induces severe lesions on susceptible wheat varieties [13,14]. Subsequently, *ToxA* and an associated 11 kb DNA region were also discovered in *Parastagonospora nodorum*, another wheat pathogen, which exhibits greater sequence diversity in *ToxA* compared to *P. tritici-repentis* [15]. This observation suggests that *ToxA* was horizontally transferred from *P. nodorum* to *P. tritici-repentis*, a hypothesis supported by the historical precedence of *P. nodorum* as a wheat pathogen [16]. Moreover, *ToxA* has been identified in *Bipolaris sorokiniana*, yet another wheat pathogen [17]. While all three species belong to the fungal order Pleosporales, they are relatively distant relatives, with their divergence occurring several million years ago [18]. This underscores the remarkable nature of HGT in shaping the evolutionary trajectory of these pathogens.

The horizontal transfer of *ToxA* between these species is thought to have been mediated by a Class II transposable element, now referred to as *ToxhAT* [19]. This 14 kb, single-copy Class II transposon is highly conserved across the three species, with ~92% nucleotide sequence similarity, indicating a recent HGT event [17]. While *ToxhAT* appears inactive in *P. tritici-repentis* and *P. nodorum* due to repeat-induced point (RIP) mutations that disrupt its terminal inverted repeats (TIRs), it remains largely intact in *B. sorokiniana*, likely due to minimal RIP activity [19,20].

Regions homologous to the DNA flanking *ToxhAT*, extending 61.2 kb upstream and 1.7 kb downstream, are shared between *P. tritici-repentis* and *P. nodorum*, suggesting that the horizontal transfer may have involved more than just *ToxhAT* [19]. Despite this, questions remain about the sequence of acquisition events and the origins of both *ToxhAT* and *ToxA* [21]. Notably, Gluck-Thaler et al. have identified a novel class of transposases termed “Starships,” characterized by a DUF3435-containing gene, which are capable of mobilizing large transposons [12]. Further work by Bucknell et al. revealed that the smaller *ToxhAT* transposon has been independently captured by two different Starships, namely *Sanctuary* in *B. sorokiniana* and *Horizon* in *P. tritici-repentis* and *P. nodorum* [22].

Although each of these species possesses additional pathogenicity mechanisms, the acquisition of *ToxA* is hypothesized to have played a pivotal role in their emergence as major pathogens of wheat. Understanding the mechanisms behind *ToxA* transfer and its retention within active transposons is therefore of paramount importance for the future management of these diseases. While *ToxA* has been well documented in *Bipolaris sorokiniana*, *Parastagonospora nodorum*, and *Pyrenophora tritici-repentis*, it has not yet been identified in the related Dothideomycetes, *Alternaria ventricosa*. *A. ventricosa* was first discovered as a new species on imported Ya Li pear fruit from China [23] and has also been isolated from wheat grains [24].

In this study, we report the discovery of *ToxA* in *Alternaria ventricosa*, marking its presence in this species for the first time. We compare this finding with other known species to further explore the mobility of *ToxA*, its associated transposon *ToxhAT*, and Starship elements. Our research provides valuable insights into the mechanisms underlying HGT and the evolutionary history of these transfer events, contributing to a better understanding of fungal adaptability and virulence.

## 2. Materials and Methods

### 2.1. Dataset

To explore the distribution of the *ToxA* gene across a broad range of fungal species, we analyzed a comprehensive dataset of 2137 whole-genome assemblies, which were sourced from the NCBI Reference Sequence Database as of July 2023. This dataset includes a diverse array of fungal genomes, with 1510 *Ascomycetes*, 476 *Basidiomycetes*, and 151 genomes from other fungal groups, providing a robust foundation for comparative analysis across multiple phylogenetic lineages. For detailed information on the specific species and genome assemblies used in this study, refer to Table S1.

### 2.2. Gene Prediction and Annotation

Gene prediction for the fungal genomes was carried out using GeneMark-ES [25] version 4.38, a tool specifically designed for eukaryotic genome annotation, with optimizations for fungal genomes to account for their unique intron–exon structures and complex intron organization. In addition to automated gene prediction, we conducted an extensive de novo annotation of repetitive elements across all three fungal species involved in this study. Manual annotation was employed for the regions surrounding the *ToxA* gene, ensuring a detailed and accurate characterization of the genomic context. This annotation facilitated a deeper understanding of the genomic environment in which the *ToxA* virulence gene is situated, particularly with respect to nearby transposable elements and other repeat families.

### 2.3. Sequence Comparisons

Amino acid sequence alignments of the *ToxA* genes from the selected species were performed using MAFFT [26] version 7.490, with default settings applied to ensure high accuracy and consistency in alignment quality. Following the alignment, a gene tree was constructed using IQ-TREE [27] version 2.0.3, utilizing its maximum likelihood (ML) framework to infer evolutionary relationships between *ToxA* sequences across the studied fungal species. Whole-genome average nucleotide identity (ANI) comparisons were carried out using pyANI version 0.2.9 software, enabling a quantitative assessment of genome-wide similarity between species containing *ToxA*. To evaluate the statistical significance of the horizontal gene transfer inference based on sequence similarity, we compared the observed genetic distances of the *ToxA* genes with the overall genome-wide ANI between the species. Assuming a Gaussian distribution of pairwise genetic distances, we calculated an exclusion range defined as μ ± 3σ, where μ is the mean and σ is the standard deviation of the genetic distances. Any similarity exceeding this threshold is considered statistically significant. The sequence similarity between *ToxA* genes observed in this study exceeded this threshold, providing strong statistical evidence supporting the HGT hypothesis. Scaffolds or chromosomes containing the *ToxA* gene were aligned across species using Progressive Mauve v2.3.1 [28], providing a comprehensive overview of synteny and structural variation in the genomic regions harboring *ToxA*. For the alignment of Starship transposons, visualizations were created using NGenomeSyn version 1.41 [29], which allowed for the identification and comparison of structural variants and insertion sites of mobile genetic elements.

### 2.4. Phylogenetic Tree of Species and HGT Detection

To assess genome completeness and ensure the inclusion of high-quality data, we used BUSCO v5.2.0 with default parameters, based on the presence or absence of 6641 predefined orthologs from the Pleosporales order (pleosporales_odb10 database). This quality control step was crucial for generating a robust phylogenomic dataset. A phylogenomic data matrix was constructed using the 6641 single-copy orthologs identified from representative species of Pleosporales. Each gene was aligned using MAFFT [26] version 7.490, employing default options to ensure high-quality alignments. Ambiguous regions within each alignment were trimmed using trimAl version 1.4 with the ‘gappyout’ option, further refining the data to focus on confidently aligned sequences.

The concatenated amino acid alignments of these BUSCO genes, each with greater than 50% taxon occupancy, were then used to build a comprehensive phylogenomic dataset. Phylogenetic analyses were performed using IQ-TREE [27] version 2.0.3, leveraging its maximum likelihood framework, which is well suited for large-scale phylogenomic datasets. The resulting phylogenetic tree, constructed in Newick format, was visualized using FigTree v1.4.0 and was drawn with a midpoint root to provide a balanced representation of evolutionary relationships (http://tree.bio.ed.ac.uk/software/figtree/ accessed on 1 May 2024). The majority of bootstrap values are 100%, indicating strong support for the tree’s topology.

To detect potential HGT events involving the *ToxA* gene, reconciliation analyses were conducted using RANGER-DTL (v2.0) [30], a specialized tool designed to infer gene duplication, loss, and transfer events. This method compares the *ToxA* gene tree with the species tree to identify discrepancies indicative of HGT.

## 3. Results

### 3.1. A Homolog of ToxA in the A. ventricosa Genome

We identified the *ToxA* gene on a 90.4 kb scaffold within the genome of *Alternaria ventricosa* BMP 2768, an isolate collected from pear in China in 2004 (NCBI BioSample: SAMN18144513). This discovery was made following an extensive analysis of 2137 species-representative fungal genomes. The genome of *A. ventricosa* was 34.7 Mbp in size, comparable to that of other species in the genus, such as *A. alternata* (33.0 Mbp), a common pathogen in a variety of natural food products. The *A. ventricosa* genome contains 12,891 predicted protein-coding genes, identified using GeneMark. To ensure the accuracy of our findings and rule out the possibility of contamination or errors, we reassembled the raw data from the FASTQ file using the SPAdes genome assembler. Upon examining the sequencing depth across the assembled contigs, we found that the scaffold containing the *ToxA* gene exhibited a depth consistent with that of other scaffolds, confirming the integrity and reliability of this genomic region.

**Figure 1:**
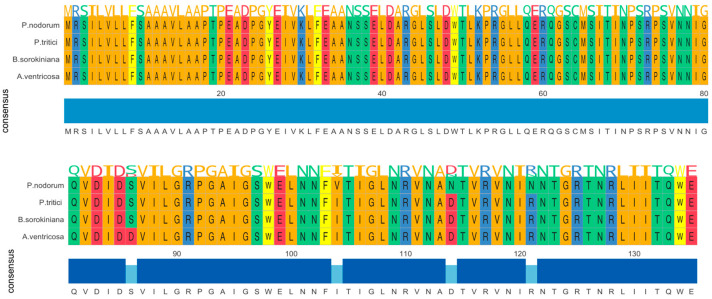
Amono acid sequence alignment of *ToxA* genes from *Alternaria ventricosa*, *Bipolaris sorokiniana*, *Parastagonospora nodorum*, and *Pyrenophora tritici-repentis*. The alignment underscores the evolutionary relationships among these fungal pathogens and suggests that horizontal gene transfer of *ToxA* may have occurred between species of Pleosporales.

The *ToxA* gene in *A. ventricosa* shows remarkable sequence similarity to *ToxA* genes in *Parastagonospora nodorum* (98.0%), *Bipolaris sorokiniana* (99.5%), and *Pyrenophora tritici-repentis* (99.5%). Amino acid sequence alignment (Figure 1) reveals only four amino acid differences among these species. Whole-genome comparisons further indicated average nucleotide identity (ANI) values of 84.0%, 83.9%, and 84.0% with *P. nodorum, B. sorokiniana*, and *P. tritici-repentis*, respectively. Importantly, the identity between the *ToxA* genes and the ANI values showed a statistically significant difference (*t*-test, *p* < 0.01), emphasizing the distinct evolutionary processes of these genes compared to the overall genome sequences.

To further assess the significance of the observed similarity, we applied a statistical approach assuming a Gaussian distribution of pairwise genetic distances, with an exclusion range of μ ± 3σ. Based on this analysis, the similarity of 98% between the *ToxA* genes falls outside the μ + 3σ range, indicating that this high similarity is much higher than would be expected from the overall genome-wide similarity (*p* < 0.01). This further supports the hypothesis that the *ToxA* gene likely underwent horizontal gene transfer (HGT) between these species.

Importantly, no other known proteins showed significant similarity to *ToxA* (E-value < 0.00001), further underscoring the uniqueness of this gene. The high sequence similarity among *ToxA* genes from *A. ventricosa*, *B. sorokiniana*, *P. nodorum*, and *P. tritici-repentis* suggests a recent common ancestry, emphasizing the crucial role of horizontal gene transfer in the spread of this virulence factor across fungal species.

### 3.2. The 14 kbp ToxhAT Transposon in the A. ventricosa Genome

**Figure 2:**
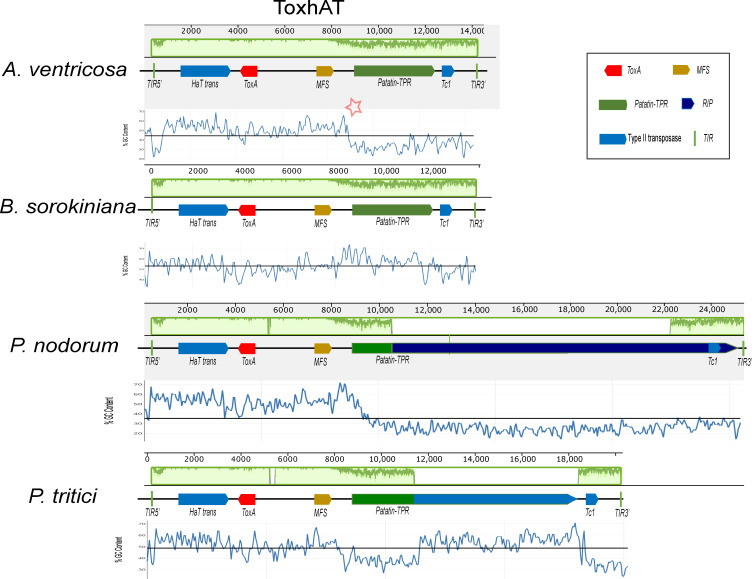
Alignment of the *ToxhAT* region from *Alternaria ventricosa*, *Pyrenophora tritici-repentis*, *Parastagonospora nodorum*, and *Bipolaris sorokiniana*, generated using Mauve version 2.4.0. In the *A. ventricosa* genome, specific regions where AT richness increases are indicated by asterisks, highlighting the variation in sequence composition at the boundaries of the conserved 14 kb region. In the context of the Mauve alignment, this plot highlights the locally collinear blocks (LCBs) that are conserved across the genomes of the species being compared. Mauve is a multiple-genome alignment tool that identifies conserved genomic regions (LCBs) and visualizes them in the plot. The LCBs are typically aligned with high sequence similarity, and their conservation is highlighted in green. The green plot represents the regions of sequence conservation across the different species. The presence of Major Facilitator Superfamily (MFS) transporters, Patatin-like TPR domains (Patatin-TPR), and repeat-induced point (RIP) mutations is highlighted within the figure, as they are key to understanding the functional context of the *ToxhAT* region. The presence of terminal inverted repeats (TIRs) marks the ends of the transposon, providing further insight into its potential mobility across the genome. This alignment sheds light on the genomic architecture of *ToxhAT* and its potential evolutionary history.

A conserved 14 kb genomic region, referred to as *ToxhAT*, was identified in *A. ventricosa* and compared across *B. sorokiniana*, *P. nodorum*, and *P. tritici-repentis* (Figure 2). Manual annotation of *ToxhAT* in each species revealed nucleotide identities of 93.5%, 94.8%, and 94.4% for *A. ventricosa* in comparison with *B. sorokiniana*, *S. nodorum*, and *P. tritici-repentis*, respectively. This 14 kb region aligns precisely with the region previously identified as horizontally transferred from *P. nodorum* to *P. tritici-repentis*, further supporting the hypothesis of HGT due to the striking nucleotide similarity across such a substantial DNA fragment.

Despite the high sequence identity within this 14 kb segment, there are key differences between the species, particularly toward the boundaries of the conserved region. As sequence similarity diminishes near the edges, there is a corresponding increase in AT richness. This pattern of increased AT content is predominantly due to the activity of repeat-induced point (RIP) mutations, particularly in *P. nodorum* and *P. tritici-repentis*. RIP, a fungal genome defense mechanism, targets repetitive elements and introduces mutations that degrade their functionality. These points of increased AT richness are highlighted for *A. ventricosa* in Figure 2 with asterisks, indicating potential RIP activity in this region as well.

### 3.3. Starship Elements Present in the A. ventricosa Genome

**Figure 3:**
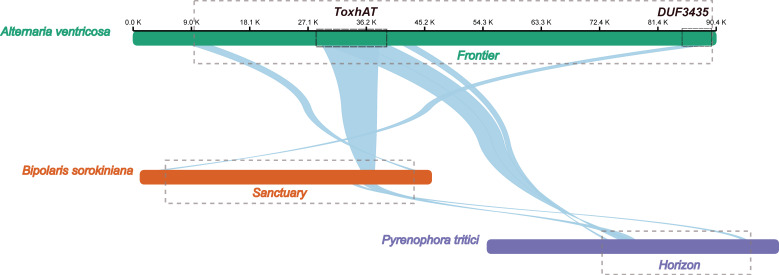
Alignment of the *ToxA*-containing 90.4 kb scaffold from the *A. ventricosa* genome with the *ToxA*-containing regions from *Bipolaris sorokiniana* strain BRIP10943a (~200 kb), and *Pyrenophora tritici-repentis* 1C-BFP (~200 kb) using NGenomeSyn. This figure is crucial in highlighting the structural conservation and variation within *ToxA* loci and further supports the hypothesis of horizontal gene transfer events.

The 90.4 kb scaffold containing the *ToxA* gene from the *A. ventricosa* genome was aligned with the *ToxA*-containing regions from *Bipolaris sorokiniana* strain BRIP10943a (~200 kb), which includes the 196 kb *Sanctuary* element, and *Pyrenophora tritici-repentis* 1C-BFP (~200 kb), which includes the 143 kb Horizon element, using NGenomeSyn (Figure 3). The longest conserved collinear block spanned the 14 kb *ToxhAT* region. Notably, outside of the *ToxhAT* region, there was almost no significant sequence similarity between the *Sanctuary* and *Horizon* elements, underscoring the uniqueness of these regions.

Interestingly, the first gene located at the 3′ end of the *A. ventricosa* scaffold was predicted to encode a DUF3435 domain protein. Alignment of the DUF3435 “captain” gene with *B. sorokiniana* revealed a high sequence identity of 98.1%. Further alignment of the 90.4 kb scaffold with *Sanctuary* showed that the first 3.8 kb, including the captain gene, was conserved with 98.5% nucleotide identity. Additionally, the 3.0 kb at the 5′ end, containing a predicted CHROMO domain protein implicated in histone binding, potentially enabling transposable elements to target specific genomic regions [31], exhibited 86.2% identity. Analysis of the genes within this transposon suggests that it is also a Starship element, sharing key structural features such as the DUF3435 tyrosine recombinase “captain” and ankyrin repeats (PF12796 and PF00023). Given these similarities, this newly identified Starship element, spanning 81.9 kb between the captain domain protein and the CHROMO domain protein, was named ‘*Frontier*’. However, beyond these conserved regions and homologous genes, *Frontier* shared limited sequence similarity with *Sanctuary* and *Horizon*. This conservation of terminal regions, along with the captain and CHROMO domain protein, may represent the minimal unit required for transposition.

A BLASTn search revealed that *Frontier* is not present in any species outside of *A. ventricosa*, highlighting its unique distribution. Comparative analysis across all three fungal species known to carry *ToxhAT* indicates that this transposon was independently captured by three distantly related Starships—*Frontier* in *A. ventricosa*, *Sanctuary* in *B. sorokiniana*, and *Horizon* in *P. tritici-repentis*. These findings underscore the complex evolutionary dynamics of Starship transposons and their role in horizontal gene transfer among fungi.

### 3.4. Interspecies HGT Events of ToxA

To explore the potential role of HGT in the discontinuous distribution of the *ToxA* gene across fungal species, we employed established methodologies, particularly focusing on reconciliation analysis using the RANGER-DTL program. This program is specifically designed to infer HGT, gene duplication, and loss events by reconciling discrepancies between gene trees and species trees.

**Figure 4:**
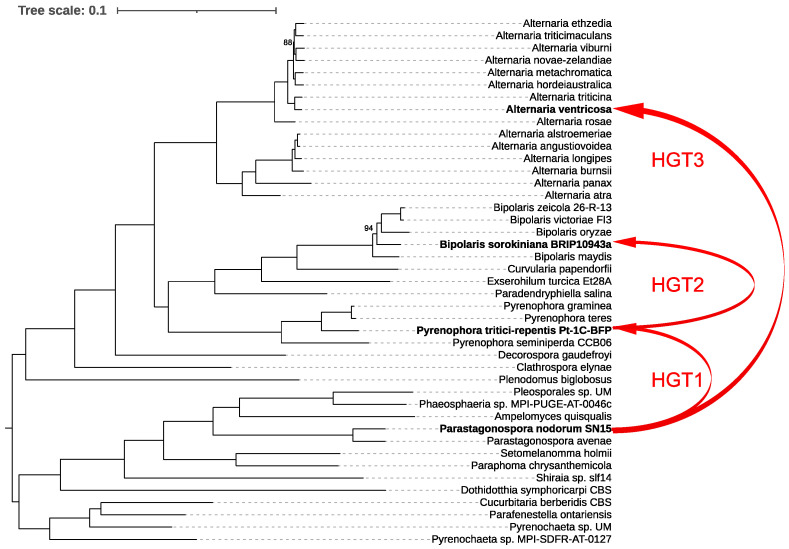
Phylogenetic relationships and horizontal gene transfer (HGT) of *ToxA* among 43 representative species within the order Pleosporales. The phylogenetic tree was constructed using IQ-TREE based on a comprehensive alignment of 6641 conserved monocore genes. The schematic representation highlights the inferred horizontal transfers of the *ToxA* gene between fungal species, as indicated by the red lines mapped onto the phylogenetic tree. These red lines illustrate all detected HGT events of *ToxA* across the species, emphasizing the complex and widespread nature of these transfers within Pleosporales. Bootstrap values below 100% are shown; values of 100% were omitted for clarity.

For our analysis, we first constructed a species tree by examining 43 representative species within the order Pleosporales, providing a robust phylogenetic framework. We then generated a *ToxA* gene tree and reconciled it with the species tree using RANGER-DTL, which revealed three distinct interspecies HGT events (Figure 4).

The first event involved the transfer of *ToxA* from *Parastagonospora nodorum* to *Pyrenophora tritici-repentis*. Following this, *P. tritici-repentis* subsequently transferred *ToxA* to *Bipolaris sorokiniana*. The third HGT event identified was the transfer of *ToxA* from *P. nodorum* to *Alternaria ventricosa*. These findings underscore the complex evolutionary history of *ToxA* and highlight the significant role of HGT in shaping the pathogenic potential of these fungal species.

## 4. Discussion

*ToxA* has been demonstrated previously to play a dominant role in the wheat diseases caused by *B. sorokiniana*, *P. nodorum*, and *P. tritici-repentis* [15]. Our study extends this understanding by identifying *ToxA* in *Alternaria ventricosa*, a species not previously associated with wheat pathogenicity. This discovery underscores the broad role of *ToxA* in facilitating disease across a diverse range of fungal pathogens. Notably, the *ToxA* gene is part of a mobile “pathogenicity element” within these fungi, suggesting that this genetic region plays a key role in pathogen adaptability and virulence. The high mobility of *ToxA*, supported by its location within a transposon-rich region, raises the possibility of its rapid dissemination across fungal species, thereby enhancing their pathogenic potential on wheat and possibly other crops.

*Alternaria* is a ubiquitous fungal genus that includes saprobic, endophytic, and pathogenic species [32]. It is associated with a wide variety of substrates including seeds, plants, agricultural products, animals, soil, and the atmosphere. Species of *Alternaria* are known as serious plant pathogens, causing major losses in a wide range of crops [32]. Several taxa are also important postharvest pathogens, causative agents of phaeohyphomycosis in immuno-compromised patients, or airborne allergens. *Alternaria ventricosa* is increasingly recognized as a potential plant pathogen, and the presence of the *ToxA* gene further suggests its role in pathogenicity. This broad ecological distribution and the presence of *ToxA* in a new species of *Alternaria* highlight the complex evolutionary dynamics of fungal virulence and the potential for *A. ventricosa* to impact agriculture in ways not previously anticipated.

Despite the high sequence similarity across the conserved 14 kb region, notable differences exist between the four species. As sequence similarity diminishes towards the edges of this region, there is a corresponding increase in AT richness, primarily driven by the activity of repeat-induced point (RIP) mutations in *P. nodorum* and *P. tritici-repentis*. RIP, a well-characterized fungal genome defense mechanism, protects against the proliferation of repetitive elements [20]. During meiosis, the RIP machinery identifies invasive repetitive DNA and introduces CpG to CpA mutations, which often leads to the insertion of stop codons in coding regions, thereby disrupting transposon activity [33]. The increase in AT content at specific loci in *Alternaria ventricosa* is highlighted by asterisks in Figure 2, reflecting its distinct sequence composition relative to the other species. However, the exact role of *ToxhAT* as an active transposon in *A. ventricosa* remains speculative, warranting further investigation into its potential mobilization and the functional dynamics of this genomic region.

Previous studies have suggested multiple HGT events for *ToxA* and the surrounding 14 kb region, known as the *ToxhAT* transposon [15]. *ToxhAT*, a smaller transposon, has been independently captured by two distinct Starships, *Sanctuary* in *B. sorokiniana* and *Horizon* in *P. tritici-repentis* and *P. nodorum* [22]. In this study, we identified the nesting of *ToxhAT* within a larger putative 81.9 kb Starship transposon, which we named *Frontier*. Unlike *Sanctuary* and *Horizon*, *Frontier* is not present in other *ToxA*-containing species. While all three Starships—*Frontier*, *Sanctuary*, and *Horizon*—carry *ToxhAT*, they share minimal sequence similarity outside this region, indicating that *ToxhAT* was independently captured by each Starship. The mechanisms driving the ‘capture’ of cargo genes like *ToxhAT* into Starships remain poorly understood. Nevertheless, the presence of this key virulence factor within three distinct Starships adds to the growing body of evidence suggesting that Starships facilitate HGT between fungi, and that the cargo they transport provides a fitness advantage to the fungal host [12,34].

The origins of *ToxhAT* remain enigmatic. Since the initial discovery of *ToxA* in the genome of *P. nodorum*, the evolutionary history of this gene has been a subject of ongoing debate [15,35,36]. *P. nodorum* currently exhibits the highest known sequence diversity for *ToxA*, lending support to the hypothesis that *ToxA* has resided in its genome for the longest period, allowing ample time for the accumulation of mutations [15,36]. The subsequent identification of *ToxA* in *B. sorokiniana* and the characterization of conserved 74 bp terminal inverted repeats across all three species strongly suggest that *ToxhAT* had a single evolutionary origin in these fungi. Our study further elucidates this evolutionary pathway, identifying three distinct interspecies HGT events (Figure 4). The first event confirms that *ToxA* was horizontally transferred from *P. nodorum* to *P. tritici-repentis*, aligning with earlier findings. This initial transfer appears to have set the stage for subsequent HGT events, where *P. tritici-repentis* transferred *ToxA* to *B. sorokiniana*. Finally, our analysis uncovered a third HGT event, where *ToxA* was transferred from *P. nodorum* to *A. ventricosa*.

These findings not only reinforce the hypothesis of a single evolutionary origin for *ToxhAT* but also highlight the dynamic and ongoing role of HGT in the dissemination of this virulence gene across different fungal species. The presence of *ToxA* in a wide variety of fungal pathogens underscores the importance of transposon-mediated HGT in facilitating rapid genetic exchange, enabling fungi to adapt quickly to new host plants and environmental pressures. In particular, transposons may act as vehicles for the mobilization of virulence genes like *ToxA*, facilitating their spread across ecologically distinct fungal populations. This phenomenon exemplifies how transposon-driven HGT can significantly contribute to the adaptive evolution of fungi, particularly in the context of host–pathogen interactions, where rapid evolution is crucial for pathogens’ survival and success.

Looking ahead, several exciting avenues for future research arise from these findings. First, unraveling the specific mechanisms behind transposon-mediated gene capture and transfer will be crucial for understanding the ecological dynamics of *ToxA* and similar virulence genes. Investigating how these transposons are mobilized, the factors that influence their acquisition by different fungal species, and the ecological conditions that favor their spread will help us better predict the emergence and spread of new pathogenic strains. Furthermore, the study of transposon activity [34] and HGT in other agriculturally important fungal species will enhance our ability to track the movement of virulence factors in fungal populations, informing disease control strategies and potentially identifying targets for novel antifungal treatments.

In addition, the integration of genomic and ecological data could help identify key environmental factors that influence the rate of HGT in fungal populations. For example, how climate change, crop rotation practices, or the use of fungicides might alter fungal gene flow and the spread of virulence factors remains an area ripe for investigation [37]. Additionally, understanding the role of transposon activity in shaping fungal genome architecture could provide new insights into the long-term evolution of fungal pathogens and their adaptability to agricultural systems.

Ultimately, by expanding our understanding of how transposons facilitate the transfer of virulence genes, this research has the potential to revolutionize strategies for managing fungal diseases. Whether by developing more effective disease-resistant crops, improving diagnostic methods for detecting virulent strains, or creating more targeted fungicides, the implications for agricultural practices and crop protection are significant. By bridging the gap between basic fungal biology, transposon evolution, and practical disease management, these insights could lead to more sustainable agricultural practices and enhanced resilience to fungal pathogens in the face of changing environmental conditions.

## Data Availability

The original contributions presented in the study are included in the article; further inquiries can be directed to the corresponding author.

## Supplementary Materials

The following supporting information can be downloaded at https://www.mdpi.com/article/10.3390/microorganisms13020376/s1. Table S1: The 2137 whole-genome assemblies analyzed in the study.

## References

[1] Huang J. (2013). Horizontal Gene Transfer in Eukaryotes: The Weak-Link Model. Bioessays.

[2] Keeling P.J., Palmer J.D. (2008). Horizontal Gene Transfer in Eukaryotic Evolution. Nat. Rev. Genet..

[3] Zhang Q., Chen X., Xu C., Zhao H., Zhang X., Zeng G., Qian Y., Liu R., Guo N., Mi W. (2019). Horizontal Gene Transfer Allowed the Emergence of Broad Host Range Entomopathogens. Proc. Natl. Acad. Sci. USA.

[4] Cao J., Liu F., Liu S., Wang J., Zhu B., Shi Y., Gao G.F. (2021). Identification of Antibiotic Resistance Genes and Associated Mobile Genetic Elements in Permafrost. Sci. China Life Sci..

[5] Liu F., Zhu Y., Yi Y., Lu N., Zhu B., Hu Y. (2014). Comparative Genomic Analysis of Acinetobacter Baumannii Clinical Isolates Reveals Extensive Genomic Variation and Diverse Antibiotic Resistance Determinants. BMC Genom..

[6] Marcet-Houben M., Gabaldón T. (2010). Acquisition of Prokaryotic Genes by Fungal Genomes. Trends Genet..

[7] Shen X.-X., Opulente D.A., Kominek J., Zhou X., Steenwyk J.L., Buh K.V., Haase M.A.B., Wisecaver J.H., Wang M., Doering D.T. (2018). Tempo and Mode of Genome Evolution in the Budding Yeast Subphylum. Cell.

[8] Aleksander M. (2024). The Interkingdom Horizontal Gene Transfer in 44 Early Diverging Fungi Boosted Their Metabolic, Adaptive, and Immune Capabilities. Evol. Lett..

[9] Liu F., Wang S.-H., Cheewangkoon R., Zhao R.-L. (2024). Uneven Distribution of Prokaryote-Derived Horizontal Gene Transfer in Fungi: A Lifestyle-Dependent Phenomenon. mBio.

[10] Liu F., Ma X.-B., Han B., Wang B., Xu J.-P., Cao B., Ling Z.-L., He M.-Q., Zhu X.-Y., Zhao R.-L. (2024). Pan-Genome Analysis Reveals Genomic Variations during Enoki Mushroom Domestication, with Emphasis on Genetic Signatures of Cap Color and Stipe Length. J. Adv. Res..

[11] Bucknell A.H., McDonald M.C. (2023). That’s No Moon, It’s a *Starship*: Giant Transposons Driving Fungal Horizontal Gene Transfer. Mol. Microbiol..

[12] Gluck-Thaler E., Ralston T., Konkel Z., Ocampos C.G., Ganeshan V.D., Dorrance A.E., Niblack T.L., Wood C.W., Slot J.C., Lopez-Nicora H.D. (2022). Giant *Starship* Elements Mobilize Accessory Genes in Fungal Genomes. Mol. Biol. Evol..

[13] Tuori R.P., Wolpert T.J., Ciuffetti L.M. (1995). Purification and Immunological Characterization of Toxic Components from Cultures of Pyrenophora Tritici-Repentis. Mol. Plant Microbe Interact..

[14] Ciuffetti L.M., Francl L.J., Ballance G.M., Bockus W.W., Lamari L., Meinhardt S.W., Rasmussen J.B. (1998). Standardization of Toxin Nomenclature in the Pyrenophora Tritici-Repentis/Wheat Interaction. Can. J. Plant Pathol..

[15] Friesen T.L., Stukenbrock E.H., Liu Z., Meinhardt S., Ling H., Faris J.D., Rasmussen J.B., Solomon P.S., McDonald B.A., Oliver R.P. (2006). Emergence of a New Disease as a Result of Interspecific Virulence Gene Transfer. Nat. Genet..

[16] McDonald M.C., Oliver R.P., Friesen T.L., Brunner P.C., McDonald B.A. (2013). Global Diversity and Distribution of Three Necrotrophic Effectors in Phaeosphaeria Nodorum and Related Species. New Phytol..

[17] McDonald M.C., Ahren D., Simpfendorfer S., Milgate A., Solomon P.S. (2017). The Discovery of the Virulence Gene ToxA in the Wheat and Barley Pathogen Bipolaris Sorokiniana. Mol. Plant Pathol..

[18] Ohm R.A., Feau N., Henrissat B., Schoch C.L., Horwitz B.A., Barry K.W., Condon B.J., Copeland A.C., Dhillon B., Glaser F. (2012). Diverse Lifestyles and Strategies of Plant Pathogenesis Encoded in the Genomes of Eighteen Dothideomycetes Fungi. PLoS Pathog..

[19] McDonald M.C., Taranto A.P., Hill E., Schwessinger B., Liu Z., Simpfendorfer S., Milgate A., Solomon P.S. (2019). Transposon-Mediated Horizontal Transfer of the Host-Specific Virulence Protein ToxA between Three Fungal Wheat Pathogens. mBio.

[20] Galagan J.E., Selker E.U. (2004). RIP: The Evolutionary Cost of Genome Defense. Trends Genet..

[21] Ghaderi F., Sharifnabi B., Javan-Nikkhah M., Brunner P.C., McDonald B.A. (2020). SnToxA, SnTox1, and SnTox3 Originated in Parastagonospora Nodorum in the Fertile Crescent. Plant Pathol..

[22] Bucknell A., Wilson H.M., Gonçalves Do Santos K.C., Simpfendorfer S., Milgate A., Germain H., Solomon P.S., Bentham A., McDonald M.C. (2024). *Sanctuary*: A *Starship* Transposon Facilitating the Movement of the Virulence Factor ToxA in Fungal Wheat Pathogens. bioRxiv.

[23] Roberts R.G., Roberts R.G. (2007). Two New Species of Alternaria from Pear Fruit. Mycotaxon.

[24] Fadhil W.F., Al-Saadoon A.H., Al-Moussawi F.M. (2022). New Records of Mycobiota Associated with Stored Wheat and Its By- Products in Iraq. Biodiversitas.

[25] Ter-Hovhannisyan V., Lomsadze A., Chernoff Y.O., Borodovsky M. (2008). Gene Prediction in Novel Fungal Genomes Using an Ab Initio Algorithm with Unsupervised Training. Genome Res..

[26] Katoh K., Standley D.M. (2013). MAFFT Multiple Sequence Alignment Software Version 7: Improvements in Performance and Usability. Mol. Biol. Evol..

[27] Minh B.Q., Schmidt H.A., Chernomor O., Schrempf D., Woodhams M.D., von Haeseler A., Lanfear R. (2020). IQ-TREE 2: New Models and Efficient Methods for Phylogenetic Inference in the Genomic Era. Mol. Biol. Evol..

[28] Darling A.C.E., Mau B., Blattner F.R., Perna N.T. (2004). Mauve: Multiple Alignment of Conserved Genomic Sequence with Rearrangements. Genome Res..

[29] He W., Yang J., Jing Y., Xu L., Yu K., Fang X. (2023). NGenomeSyn: An Easy-to-Use and Flexible Tool for Publication-Ready Visualization of Syntenic Relationships across Multiple Genomes. Bioinformatics.

[30] Bansal M.S., Kellis M., Kordi M., Kundu S. (2018). RANGER-DTL 2.0: Rigorous Reconstruction of Gene-Family Evolution by Duplication, Transfer and Loss. Bioinformatics.

[31] Kordis D. (2005). A Genomic Perspective on the Chromodomain-Containing Retrotransposons: Chromoviruses. Gene.

[32] Woudenberg J.H., Groenewald J.Z., Binder M., Crous P.W. (2013). Alternaria Redefined. Stud. Mycol..

[33] Selker E.U. (1990). Premeiotic Instability of Repeated Sequences in Neurospora Crassa. Annu. Rev. Genet..

[34] Urquhart A.S., Vogan A.A., Gardiner D.M., Idnurm A. (2023). *Starships* Are Active Eukaryotic Transposable Elements Mobilized by a New Family of Tyrosine Recombinases. Proc. Natl. Acad. Sci. USA.

[35] Ciuffetti L.M., Manning V.A., Pandelova I., Betts M.F., Martinez J.P. (2010). Host-Selective Toxins, Ptr ToxA and Ptr ToxB, as Necrotrophic Effectors in the Pyrenophora Tritici-Repentis-Wheat Interaction. New Phytol..

[36] Stukenbrock E.H., McDonald B.A. (2007). Geographical Variation and Positive Diversifying Selection in the Host-Specific Toxin SnToxA. Mol. Plant Pathol..

[37] Singh B.K., Delgado-Baquerizo M., Egidi E., Guirado E., Leach J.E., Liu H., Trivedi P. (2023). Climate Change Impacts on Plant Pathogens, Food Security and Paths Forward. Nat. Rev. Microbiol..

